# Advancing Privacy-Preserving Health Care Analytics and Implementation of the Personal Health Train: Federated Deep Learning Study

**DOI:** 10.2196/60847

**Published:** 2025-02-06

**Authors:** Ananya Choudhury, Leroy Volmer, Frank Martin, Rianne Fijten, Leonard Wee, Andre Dekker, Johan van Soest

**Affiliations:** 1 GROW Research Institute for Oncology and Reproduction Maastricht University Medical Center+ Maastricht Netherlands; 2 Clinical Data Science Maastricht University Maastricht Netherlands; 3 Netherlands Comprehensive Cancer Organization (IKNL) Eindhoven Netherlands; 4 Brightlands Institute for Smart Society (BISS) Faculty of Science and Engineering (FSE) Maastricht University Heerlen Netherlands

**Keywords:** gross tumor volume segmentation, federated learning infrastructure, privacy-preserving technology, cancer, deep learning, artificial intelligence, lung cancer, oncology, radiotherapy, imaging, data protection, data privacy

## Abstract

**Background:**

The rapid advancement of deep learning in health care presents significant opportunities for automating complex medical tasks and improving clinical workflows. However, widespread adoption is impeded by data privacy concerns and the necessity for large, diverse datasets across multiple institutions. Federated learning (FL) has emerged as a viable solution, enabling collaborative artificial intelligence model development without sharing individual patient data. To effectively implement FL in health care, robust and secure infrastructures are essential. Developing such federated deep learning frameworks is crucial to harnessing the full potential of artificial intelligence while ensuring patient data privacy and regulatory compliance.

**Objective:**

The objective is to introduce an innovative FL infrastructure called the Personal Health Train (PHT) that includes the procedural, technical, and governance components needed to implement FL on real-world health care data, including training deep learning neural networks. The study aims to apply this federated deep learning infrastructure to the use case of gross tumor volume segmentation on chest computed tomography images of patients with lung cancer and present the results from a proof-of-concept experiment.

**Methods:**

The PHT framework addresses the challenges of data privacy when sharing data, by keeping data close to the source and instead bringing the analysis to the data. Technologically, PHT requires 3 interdependent components: “tracks” (protected communication channels), “trains” (containerized software apps), and “stations” (institutional data repositories), which are supported by the open source “Vantage6” software. The study applies this federated deep learning infrastructure to the use case of gross tumor volume segmentation on chest computed tomography images of patients with lung cancer, with the introduction of an additional component called the secure aggregation server, where the model averaging is done in a trusted and inaccessible environment.

**Results:**

We demonstrated the feasibility of executing deep learning algorithms in a federated manner using PHT and presented the results from a proof-of-concept study. The infrastructure linked 12 hospitals across 8 nations, covering 4 continents, demonstrating the scalability and global reach of the proposed approach. During the execution and training of the deep learning algorithm, no data were shared outside the hospital.

**Conclusions:**

The findings of the proof-of-concept study, as well as the implications and limitations of the infrastructure and the results, are discussed. The application of federated deep learning to unstructured medical imaging data, facilitated by the PHT framework and Vantage6 platform, represents a significant advancement in the field. The proposed infrastructure addresses the challenges of data privacy and enables collaborative model development, paving the way for the widespread adoption of deep learning–based tools in the medical domain and beyond. The introduction of the secure aggregation server implied that data leakage problems in FL can be prevented by careful design decisions of the infrastructure.

**Trial Registration:**

ClinicalTrials.gov NCT05775068; https://clinicaltrials.gov/study/NCT05775068

## Introduction

Federated learning (FL) allows the collaborative development of artificial intelligence models using large datasets, without the need to share individual patient-level data [1-4]. In FL, partial models trained on separate datasets are shared, but not the data itself, hence a global model is derived from the collective set of partial models. This study introduces an innovative FL framework known as the Personal Health Train (PHT) that includes the procedural, technical, and governance components needed to implement FL on real-world health care data, including the training of deep learning neural networks [5]. The PHT infrastructure is supported by a free and open-source infrastructure known as “priVAcy preserviNg federaTed leArninG infrastructurE for Secure Insight eXchange,” that is, Vantage6 [6]. We will describe in detail an architecture for training a deep learning model in a federated way with 12 institutional partners located in different parts of the world.

Sharing patient data between health care institutions is tightly regulated due to concerns about patient confidentiality and the potential for misuse of data. Data protection laws—including the European Union’s General Data Protection Regulations; Health Insurance Portability and Accountability Act of 1996 (HIPAA) in the United States; and similar regulations in China, India, Brazil, and many other countries—place strict conditions on the sharing and secondary use of patient data [7]. Incompatibilities between laws and variations in the interpretation of such laws lead to strong reluctance about sharing data across organizational and jurisdictional boundaries [8-10].

To address the challenges of data privacy, a range of approaches have been published in the literature. Differential privacy, homomorphic encryption, and FL comprise a family of applications known as “privacy enhancing technologies” [11-13]. The common goal of privacy-enhancing technologies is to unlock positively impactful societal, economic, and clinical knowledge by analyzing data en masse, while obscuring the identity of study subjects that make up the dataset. Academic institutions are more frequently setting up controlled workspaces (eg, secure research environments [SREs]), where multiple researchers can collaborate on data analysis within a common cloud computing environment, but without allowing access to the data from outside the SRE desktop; however, this assumes that all the data needed have been transferred into the SRE in the first place [14,15]. Similarly, the National Institutes of Health has set up an “Imaging Data Commons” to provide secure access to a large collection of publicly available cancer imaging data colocated with analysis tools and resources [16]. Other researchers have shown that blockchain encryption technology can be used to securely store and share sensitive medical data [17]. Blockchain ensures data integrity by maintaining an audit trail of every transaction, while zero trust principles make sure the medical data are encrypted and only authenticated users and devices interact with the network [18].

From a procedural point of view, the PHT manifesto for FL rules out the sharing of individual patient-level data between institutions, no matter if the patient data have been deidentified or encrypted [19]. The privacy-by-design principle here may be referred to as “safety in numbers,” that is, any single individual’s data values are obscured, by computing either the descriptive statistics or the partial model, over multiple patients. PHT allows sufficiently adaptable methods of model training, such as iterative numerical approximation (eg, bisection) or federated averaging (FedAvg [20]), and does not mandatorily require model gradients or model residuals, which are well-known avenues of privacy attacks [21-24]. Governance is essential with regards to compliance with privacy legislation and division of intellectual property between collaboration partners. A consortium agreement template for PHT has been made openly accessible [25], which is based on our current consortium ARGOS (artificial intelligence for gross tumor volume segmentation) [26]. Technologically, PHT requires 3 interdependent components to be installed—“tracks” are protected telecommunications channels that connect partner institutions, “trains” are Docker containerized software apps that execute a statistical analysis that all partners have agreed upon, and “stations” are the institutional data repositories that hold the patient data [23]. It is this technological infrastructure—the tracks, trains, and stations—that is supported by the aforementioned Vantage6 software, for which detailed stand-alone documentation exists [27].

The paper proposes a federated deep learning infrastructure based on the PHT manifesto [19], which provides a governance and ethical, legal, and social implications framework for conducting FL studies across geographically diverse data providers. The research aims to showcase a custom FL infrastructure using the open-source Vantage6 platform, detailing its technological foundations and implementation specifics. The paper emphasizes the significance of the implemented custom federation strategy, which maintains a strict separation between intermediate models from both internal and external user access. This approach is crucial for safeguarding the security and privacy of sensitive patient data, as it prevents potential reverse engineering of intermediate results that could compromise confidentiality. This aggregation strategy is particularly important in the case of deep learning–based studies where multiple iterations of models or gradients are necessary to derive an optimal global model.

**Figure 1:**
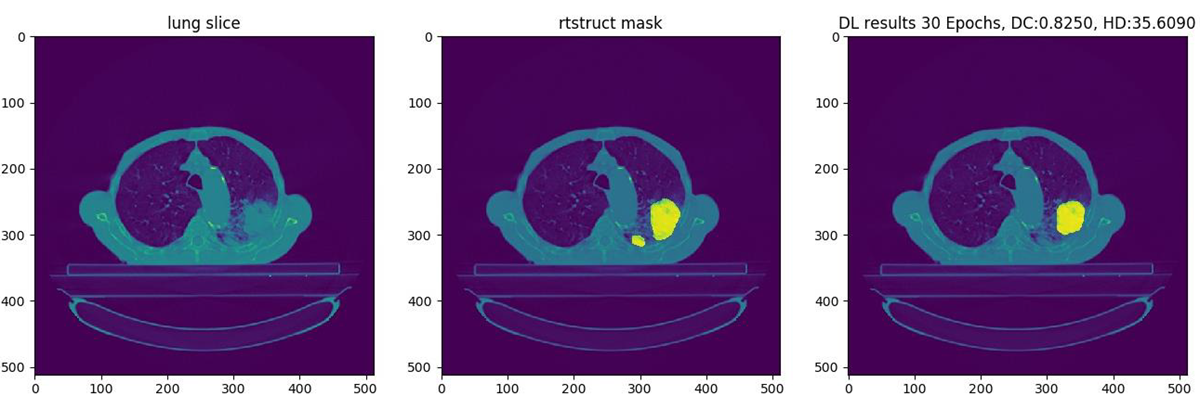
Illustrative result on a hold-out validation slice; the main bulk of the gross tumor volume as determined by the oncologist (middle) has been correctly delineated by the deep learning algorithm (right), but a small tumor mass adjacent and to the lower right of the main gross tumor volume mass has been missed (reproduced from Figure 6 of Chapter 4 of the thesis by Patil [29], which is published under the Taverne License [Article 25fa of the Dutch Copyright Act]).

To demonstrate the infrastructure’s robustness and practical applicability, the study presents a proof-of-concept involving the development of a federated deep learning algorithm based on 2D convolutional neural network (CNN) architecture [28]. This algorithm was implemented to automatically segment gross tumor volume (GTV) from lung computed tomography (CT) images of patients with lung cancer. Figure 1 [29] demonstrates a manual segmentation and deep learning–based segmentation of a tumor in the chest CT image of a patient. The subsequent sections provide a comprehensive account of the precise technical specifications of the infrastructure that links 12 hospitals across 8 nations, covering 5 continents. The algorithm developed learns from the distributed datasets and deploys it using the infrastructure. However, it is important to mention that the choice of the use case is only exemplary in nature, and the infrastructure is equipped to train any kind of deep learning architecture for relevant clinical use cases.

The research used a deep learning architecture because in recent times the application of deep learning in health care has led to impressive results, specifically in the areas of natural language processing and computer vision (medical image analysis), with the promise for more efficient diagnostics and better predictions of treatment outcomes in future [30-35]. However, for robust generalizability, and to earn clinicians’ acceptance, it is essential that artificial intelligence apps are trained on massive volumes of diverse and demographically representative health care data across multiple institutions. Given the barriers to data sharing, this is clearly an area where FL can play a vital role. Many studies have been published that present FL on medical data including federated deep learning [36-40]. However, only a limited number of studies have documented the use of dedicated frameworks and infrastructures in a transparent manner. The adoption of a custom federation strategy or absence of explicit reporting on the used infrastructure is observed in most of the studies. Table 1 summarizes the small number of FL studies that have been published in connection with deep learning investigations related to medical image segmentations to date.

The paper primarily focuses on demonstrating the training and aggregation mechanism of a deep learning architecture within a FL framework. It deliberately avoids delving into the optimization of model performance or clinical accuracy, as these aspects fall outside the paper’s scope. Instead of emphasizing the selection of an optimal CNN architecture or aggregation strategy [39], the research concentrates on elucidating the functionality of the FL infrastructure. Existing literature has shown that FL models can achieve performance comparable to centrally trained models [38,41,45-47]. This supports the assumption that, given identical datasets and CNN architectures, a model trained using FL would likely yield similar results to one trained through centralized methods. The paper operates under this premise, prioritizing the explanation of the FL process over demonstrating performance parity with centralized training approaches.

The study highlights 3 key points as follows:

- FL is particularly well suited for deep learning applications, which typically require vast amounts of data. This makes it an ideal showcase for the federated approach.
- When implementing federated deep learning, it is crucial to have a robust infrastructure and use a customized, secure aggregation strategy. These elements are essential for safeguarding the privacy of sensitive patient information.
- FL in real-world medical data is not just a technological challenge; it requires a comprehensive strategy that addresses ethical, legal, governance, and organizational aspects, as highlighted by the PHT manifesto.

**Table 1:** Existing studies from the literature focusing on federated deep learning on medical images.

Infrastructure and clinical use case			Data type		Scale
**NVIDIA FLARE/CLARA**					
	Prostate segmentation of T2-weighted MRI^a^ [41]	DICOM MRI		3 centers	
	COVID-19 pneumonia detection [42]	Chest CT^b^		7 centers	
**Tensorflow federated**					
	COVID-19 prediction from chest CT images [43]	Chest CT		3 datasets	
**OpenFL**					
	Glioblastoma tumor boundary detection [44]	Brain MRI		71 centers	

^a^MRI: magnetic resonance imaging.

^b^CT: computed tomography.

The findings of the proof-of-concept study, as well as the implications and limitations of the infrastructure and the results, are discussed. The subsequent section of the paper is structured as follows: the *Methods* section describes the approach taken, followed by the *Results*, which detail the implementation of the infrastructure and a proof-of-concept execution. Finally, the paper concludes with a *Discussion* section.

## Methods

### Overview

When conducting a federated deep learning study, it is crucial to consider several key perspectives, which include both technical as well as organizational and legal aspects. These key factors have been instrumental in designing the infrastructure architecture used for training the deep learning algorithm. In this section, we discuss the technical details while adhering to an Ethics-Legal-Social Impact framework as laid down by the PHT manifesto. The technical design decisions are based on the following assumptions:

### Data Landscape

Understanding the data landscape is crucial in designing and deploying FL algorithms. The technological approaches for handling horizontally partitioned data, where each institution contains nonoverlapping human subjects but the domain of the data (eg, CT images of lung cancer) is the same across different institutions, can differ significantly from those used for vertically partitioned data, where each institution contains the same human subjects but the domain of the data do not overlap (eg, CT scans in one, but socioeconomic metrics in another). Additionally, unstructured data, such as medical images, requires different algorithms and preprocessing techniques compared with structured data. In this paper, the architecture will only focus on CT scans and horizontally partitioned patient data.

### Data Preprocessing

In a horizontally partitioned FL setting, the key preprocessing steps can be standardized and sent to all partner institutions. However, the workflow needs to handle differences in patients, scan settings, and orientations. Anonymization, quality improvements, and DICOM standardization ensure homogeneity and high quality across hospitals. These offline preprocessing steps, applied consistently to the horizontally partitioned data, enabled using the same model across institutions, crucial for the FL study’s success.

### Network Topology of the FL Infrastructure

The network topology choice for implementing FL can vary from client-server, peer-to-peer, tree-based hierarchical, or hybrid topologies. While peer-to-peer architecture is more cost-effective and offers a high capacity, it has the disadvantages of a lack of security and privacy constraints and a complex troubleshooting process in the event of a failure. The choice of network topology for this study is based on a client-server architecture, offering a single point of control in the form of the central server.

### Choice of Model Aggregation Site

For a client-server architecture, the model aggregation can occur either in one of the data providers’ machines, the central server, or in a dedicated aggregation server. For this implementation, we opted to use a dedicated aggregation server. The details and benefits of the implementation are discussed in the next section.

### Training Strategy

The communication mechanism for transferring weights can be either synchronous, asynchronous, or semisynchronous, and weights can be consolidated using ensemble learning, FedAvg, split learning, weight transfer, or swarm learning. The strategy used for this study is based on a synchronous mechanism using the FedAvg algorithm. This gives a simple approach, where the averaging algorithm waits for all the data centers to transfer the locally trained model before initiating the averaging.

**Figure 2:**
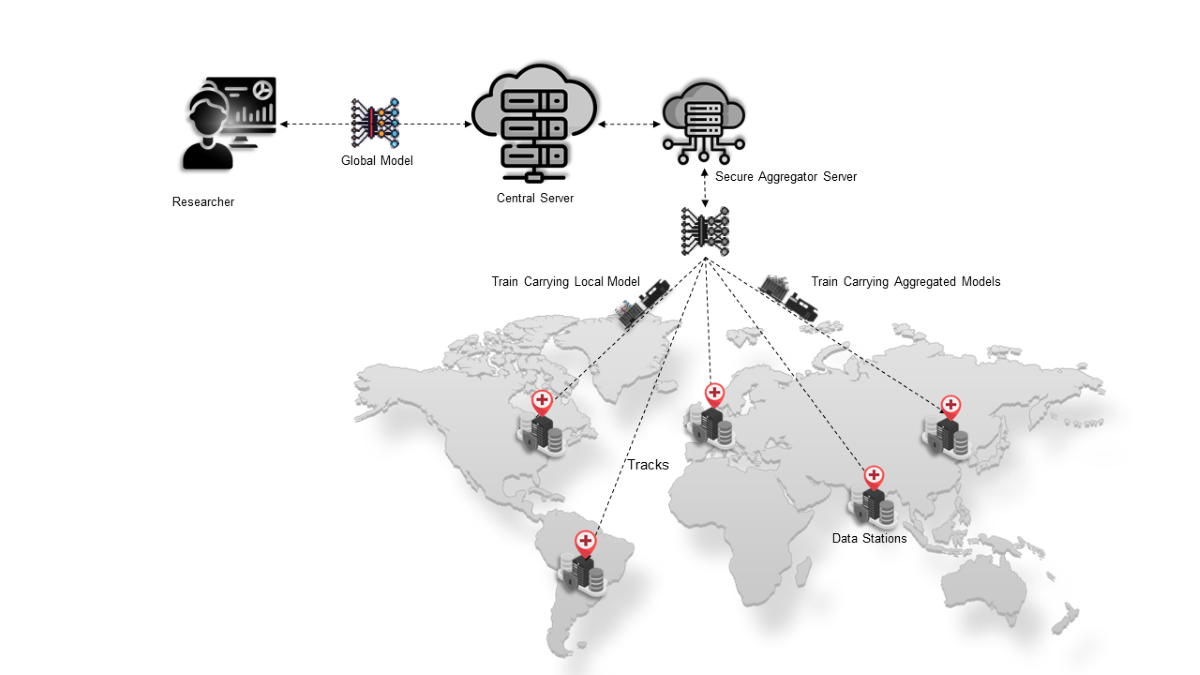
Overall architecture of ARGOS (artificial intelligence for gross tumor volume segmentation) federated deep learning architecture adapted from Vantage6. The figure depicts a researcher connected to the central server, a secure aggregation server, trains carrying models, connected data stations, and the communicating tracks.

Based on the assumption, Figure 2 depicts the overall architecture of the federated deep learning study presented in the paper. The next section describes the FL Infrastructure in detail.

### The ARGOS Federated Deep Learning Infrastructure

#### Overview

In accordance with the PHT principles, the ARGOS infrastructure is comprised of 3 primary categories of components, labeled as the data stations, the trains, and the track. Furthermore, the architectural framework encompasses various roles that map to the level of permissions and access, specifically a track provider, the data providers, and the researcher. The infrastructure implementation can be further categorized into 3 important components: a central coordination server, a secure aggregation server (SAS), and the nodes located at each “data station.” In the following sections, we attempt to describe each of these components and the respective stakeholders responsible for maintaining them.

#### Central Coordinating Server

The central coordination server is located at the highest hierarchical level and serves as an intermediary for message exchange among all other components. The components of the system, including the users, data stations, and SAS, are registered entities that possess well-defined authentication mechanisms within the central server. It is noteworthy that the central acts as a coordinator rather than a computational engine. Its primary function is to store task-specific metadata relevant to the task initiated for training the deep learning algorithm. In the original Vantage6 infrastructure, the central server also stores the intermediate results. In the ARGOS infrastructure, the central server is designed to not store any intermediate results but only the global aggregated model at the end of the entire training process.

#### Secure Aggregation Server

The SAS refers to a specialized station that contains no data and functions as a consolidator of locally trained models. The aggregator node is specifically designed to possess a Representational State Transfer (REST)–application programming interface (API) termed as the API Forwarder. The API Forwarder is responsible for managing the requests received from the data stations and subsequently routing them to the corresponding active Docker container, running the aggregation algorithm.

**Figure 3:**
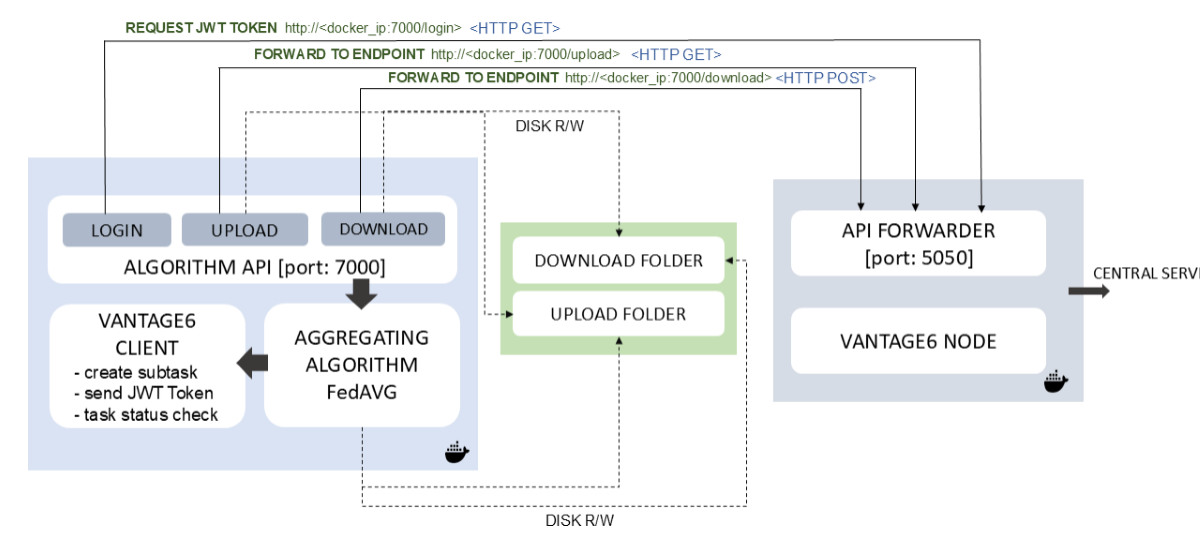
Architecture of the secure aggregation server, showing incoming and outgoing requests from the data station nodes. The upload and download folders are temporary locations used within the running Docker container to store the local and averaged models through disk read or write operations. The API forwarder, running at port 5050 and embedded within the Vantage6 infrastructure, forwards the incoming requests from the data station nodes to the algorithm API running at local port 7000 within the Docker container through HTTP requests. The SAS is hosted behind the firewall of a proxy server, which allows only hypertext transfer protocol secure (HTTPS) communication from the participating nodes. API: application programming interface; FedAvg: federated averaging; JWT: JSON Web Token.

To prevent any malicious or unauthorized communication with the aggregator node, each data station is equipped with a JSON Web Token (JWT) that is unique for each iteration. The API Forwarder only accepts communications that are accompanied by a valid JWT. The implementation of this functionality guarantees the protection of infrastructure users and effectively mitigates the risk of unauthorized access to SAS. Figure 3 shows the architecture and execution mechanism for the SAS.

#### Data Stations

**Figure 4:**
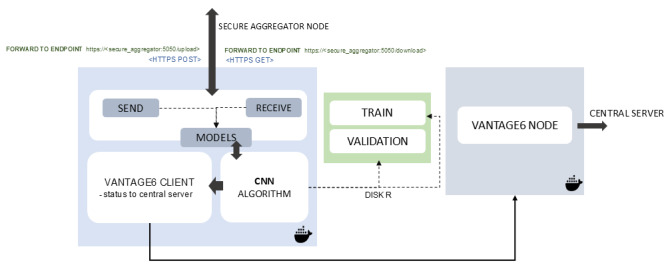
Architecture of the data station node component. The node runs the CNN algorithm to learn from the local data. The node further sends and receives model weights from the secure aggregation server. The train and validation folders are persistent locations within the data stations, storing the preprocessed NIFTI images. At the end of each training cycle, the intermediate averaged model is first evaluated on the validation sample. CNN: convolutional neural network; HTTPS: hypertext transfer protocol secure; NIFTI: neuroimaging informatics technology initiative.

Data stations are devices located within the confines of each hospital’s jurisdiction that are not reachable or accessible from external sources other than Vantage6. The data stations communicate with the central server through a pull mechanism. Furthermore, the data stations not only serve as hosts for the infrastructure node but also offer the essential computational resources required for training the deep learning network. The infrastructure node is the software component installed in the data stations that orchestrates the local execution of the model and its communication with the central server and the SAS. Each data station is equipped with at least 1 graphics processing unit (GPU), which enables the execution of CNNs. Preprocessing of the raw CT images was executed locally, using automated preprocessing scripts packaged as Docker containers, and the preprocessed CT images are stored within a file system volume in each station. The CNN Docker is designed and allowed to access the preprocessed images during training. The primary function of the data station is to receive instructions from both the SAS and the central server, perform the computations needed for training the CNN algorithm, and subsequently transmit the model weights back to the respective sources. Figure 4 depicts the architectural layout of the data station and node component of the infrastructure.

#### Train

The “train” in the form of a Docker image encompasses several components bundled together: an untrained U-Net [48,49], a type of CNN architecture designed for image segmentation tasks for training on local data; the aggregation algorithm used for consolidating the models; and a secondary Python Flask API known as the Algorithm API for facilitating the communication of these models. The Algorithm API is designed to cater to requests from the API Forwarder and is built within the algorithm container. Two levels of API ensured that the node could handle multiple requests and divert to appropriate Docker containers. Furthermore, the first level of API also helps in restricting malicious requests by checking the JWT token signature, so that the models within the master Docker container are protected. Each data station is responsible for training and transmitting the CNN model to the aggregator server. This suggests that the aggregation algorithm exhibits a waiting period during which it ensures that all data stations have effectively transmitted their models to the server before proceeding to the next iterations. The process is executed in an iterative manner until convergence is achieved or the specified number of iterations is attained.

#### Tracks and Track Provider

The various infrastructure components establish coordination among themselves through the use of secure communication channels commonly referred to as the “tracks.” The communication channels are enabled with end-to-end encryption. The responsibility for the maintenance of the infrastructure, including the hosting of the central coordinating server and the specialized SAS, lies with the track provider. The track provider is additionally accountable for the maintenance of the “tracks” and aids the data providers in establishing the local segment of the infrastructure known as the “nodes.”

#### Data Provider

Data providers refer to hospitals and health care organizations that are responsible for curating the pertinent datasets used for training the deep learning network. The responsibility of hosting the data stations within their respective local jurisdiction lies with the data provider. They exercise authority over the data as well as the infrastructure component called the node.

#### Researcher

The researcher is responsible for activating the deep learning algorithm and engaging in the authentication process with the central coordinating server using a registered username and password. This allows the researcher to establish their identity and gain secure access to the system, with their communication safeguarded through end-to-end encryption. The researcher can then assign tasks to individual nodes, monitor progress, and terminate tasks in the event of failure. Importantly, the researcher’s methodology is designed to keep the intermediate outcomes of the iterative deep learning training process inaccessible, ensuring that the ultimate global model can only be obtained upon completion of all training iterations, thereby mitigating the risk of unauthorized access by malicious researchers to the intermediate models and providing a security mechanism against insider attacks.

#### Training Process

Each of the components described above works in a coordinated manner to accomplish the convergence of the deep learning algorithm. The training process begins with the researcher authenticating with the central server. Upon successful authentication, the researcher specifies the task details, including a prebuilt Docker image, input parameters, number of iterations, and the identity of the SAS. The task is then submitted to the central server, which forwards it to the connected nodes. The SAS is the first to receive the task request. It downloads the specified Docker image from the registry and initiates the master algorithm. The master algorithm orchestrates the training at each data station node through the central server. The central server then forwards a subtask request to all the data stations. Like the SAS, the data nodes download the same Docker image and initiate the node part of the algorithm. The node algorithm runs the learning process on local data for the specified number of epochs. After each training cycle, the node algorithm sends the local model weights to the SAS.

The SAS verifies the JWT signature of each received model and forwards the request to the Algorithm API. The Algorithm API extracts the weight and metadata information of the models. Once the SAS receives all the required locally trained models for that cycle, it initiates the FedAvg algorithm to consolidate the models and create an intermediate averaged model, which is stored locally. This completes the first iteration of the training cycle. For the second and subsequent iterations, the data stations request the SAS to send the intermediate averaged model weights from the previous iteration. The SAS validates these requests and sends the model weights to the data stations, which then use them for further training on their local data. This cycle of training and averaging continues until the model converges or the desired number of iterations is reached.

**Figure 5:**
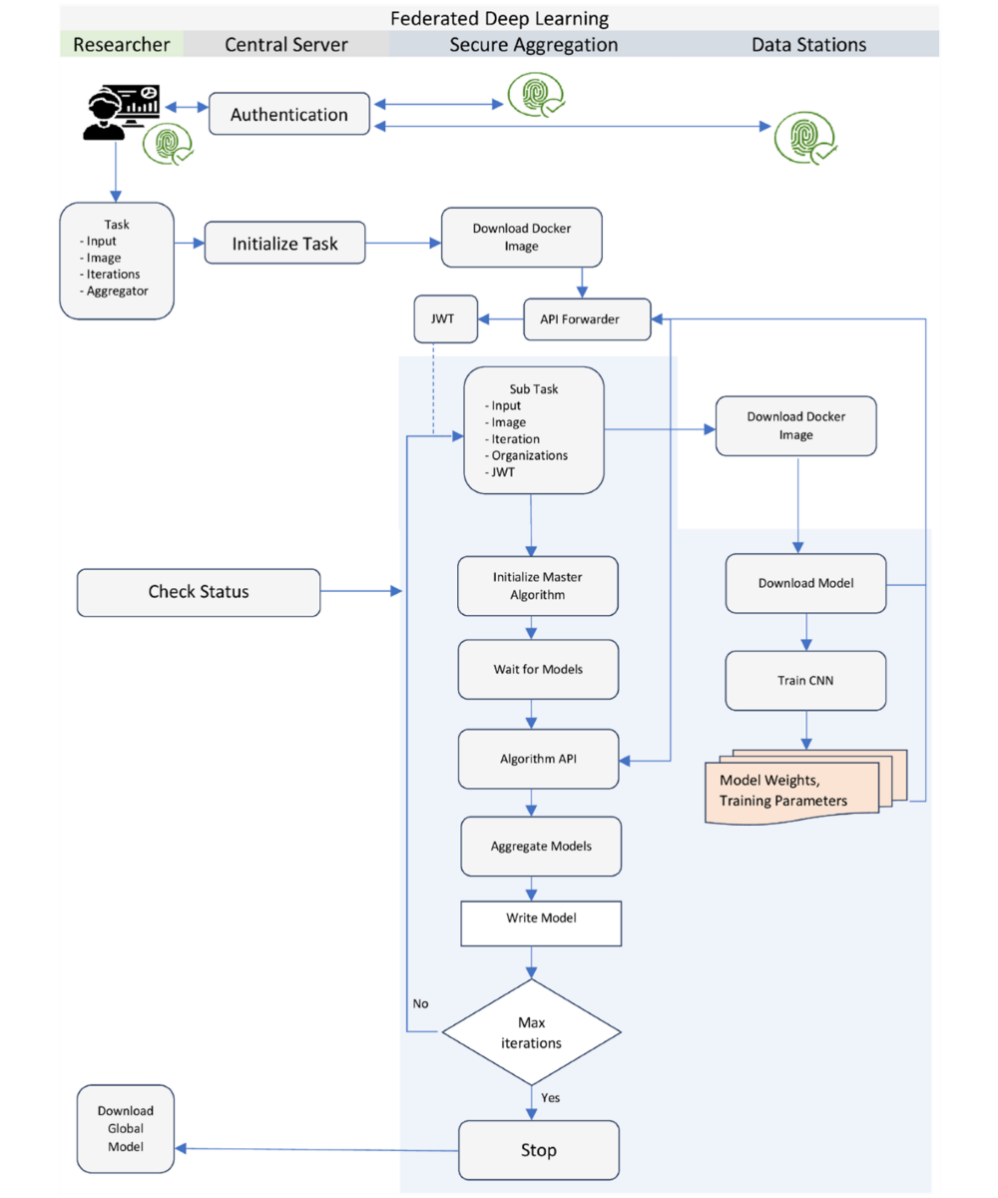
Process illustration of federated deep learning training. All entities, including the researcher, the central aggregation server, and the data stations, first authenticate with the central server. The researcher creates a task description and submits the task to the central server, which then forwards the request to the secure aggregation node to start the master task. The master task then sends a request to all data stations to download the algorithm Docker image and start training on the local data. Researchers can monitor the algorithm’s execution status on the central server using the “check status” function, which reports whether each iteration is completed or aborted as processed by the secure aggregation server and data stations. At the end of each local training, the data stations send the models to the API forwarder of the secure aggregation node by authenticating against a valid JWT token. The JWT token ensures that no unauthorized data station is able to send or receive models from the secure aggregation server. API: application programming interface; CNN: convolutional neural network; JWT: JSON Web Token.

At the end of the training process, the SAS sends a notification to the researcher indicating the successful completion of the task. The researcher can then download the final global model from the server. It is important to note that during the training iterations, the researcher or other users of the infrastructure do not have access to the intermediate averaged models generated by the SAS. This design choice prevents the possibility of insider attacks and data leakage, as users cannot regenerate patterns from the training data using the intermediate models. Figure 5 shows the diagrammatic representation of the training process spread across the infrastructure components.

#### Code Availability

The federated deep learning infrastructure and the algorithm used in this research are open source and publicly available. The codebase, encompassing the components of the infrastructure, the algorithm, and wrappers for running it in the infrastructure and the researcher notebooks, are all available and deposited on GitHub, a public repository platform, under the Apache 2.0 license. This open access allows the research community to scrutinize and leverage our implementation for further development in the field of FL.

The Vantage6 (version 2.0.0) [27,50] open-source software was customized to cater to the specific requirements for running the deep learning algorithm. The central server (Vantage6 version 2.0.0) and the aggregator server were hosted by Medical Data Works BV in 2 separate cloud machines (Microsoft Azure). At each participating center, the “node” component of the software was installed and setup either on a physical or cloud machine running Ubuntu (version 16.0) or above with an installation of Python, (version 3.7 or above; Python Software Foundation), Docker Desktop (personal edition), and NVIDIA CUDA GPU interface (version 11.0). The source code of the customized “node” [51] and setup instructions [52] are available on respective GitHub repositories. The federated deep learning algorithm was adapted to the infrastructure as Python scripts [53] and wrapped in a Docker container. Separately, the “researcher” notebooks [54] containing python scripts for connecting to the infrastructure and running the algorithms are also available on GitHub. Table 2 provides an outline of the resource requirement and computational cost of the experiment.

**Table 2:** Resource requirement and computational cost.

End points	Resource requirement		Average execution time (per iteration)
	Software	Hardware	
Central server	Ubuntu (version 16) and above Docker Desktop Python (3.7 or above) Vantage6 (version 2.0.0)	4 CPUsa 16 GB RAM 20 GB Disk Space	N/A^b^
Data station	Ubuntu (version 16) and above Docker Desktop Python (3.7 or above) Vantage6 (version 2.0.0) CUDA GPU Interface (version 11.0)	4 CPUs 1 GPUc 16 GB RAM 40 GB disk space	40 mins
Secure aggregation server	Ubuntu (version 16) and above Docker Desktop Python (3.7 or above) Vantage6 (version 2.0.0)	4 CPUs 16 GB RAM 40 GB disk space	60 seconds

^a^CPU: central processing unit.

^b^Not applicable.

^c^GPU: graphics processing unit.

### Ethical Considerations

The work was performed independently with the ethics board’s approval from each participating institution. Approvals from each of the participating institutions including soft copies of approval have been submitted to the leading partner. The lead partner’s institutional review board approval (MAASTRO Clinic, The Netherlands) is “W 20 11 00069” (approved on November 24, 2020). The authors attest that the work was conducted by the ethical standards of the responsible committee on human experimentation (institutional and national) and with the Helsinki Declaration of 1975.

## Results

### Overview

The study was carried out and concluded in 4 primary stages using an agile approach as follows: planning, design and development, partner recruitment, and execution of federated deep learning. The planning phase of the study, which encompassed a meticulous evaluation and determination of the following inquiries, held equal significance to the description of the clinical issue and data requirements.

- What are the minimum resource requirements for each participating center?
- How to design a safe and robust infrastructure to effectively address the requirements of a federated deep learning study?
- How can a reliable and data-agnostic federated deep learning algorithm be designed?
- What are the operational and logistical challenges associated with conducting a large-scale federated deep learning study?

The second phase, that is, the design and development phase, primarily focused on the creation, testing, and customization of the Vantage6 infrastructure for studies specifically focused on deep learning. To meet the security demands of these investigations, this study involved the development of the SAS, which was not originally included in the Vantage6 architecture. The CNN algorithm was packaged as a Docker container and made compatible with the Vantage6 infrastructure, allowing it to be easily deployed and used within the Vantage6 ecosystem. Prior to the deployment of the algorithm, it underwent testing using multiple test configurations consisting of data stations that were populated with public datasets.

The primary objective of the third phase entailed the recruitment of partners who displayed both interest and suitability from various global locations. The project consortium members became part of the project by obtaining the necessary institutional review board approvals and signing an infrastructure user agreement. This agreement enabled them to install the required infrastructure locally and carry out algorithmic execution. The inclusion criteria for patient data, as well as the technology used for data anonymization and preprocessing, were provided to each center. The team collaborated with each partner center to successfully implement the local component of the infrastructure.

The concluding stage of the study involved the simultaneous establishment of connections between all partner centers and the existing infrastructure. The algorithm was subsequently initiated by the researcher and the completion of the predetermined set of federated iterations was awaited across all centers.

### Proof of Concept

**Figure 6:**
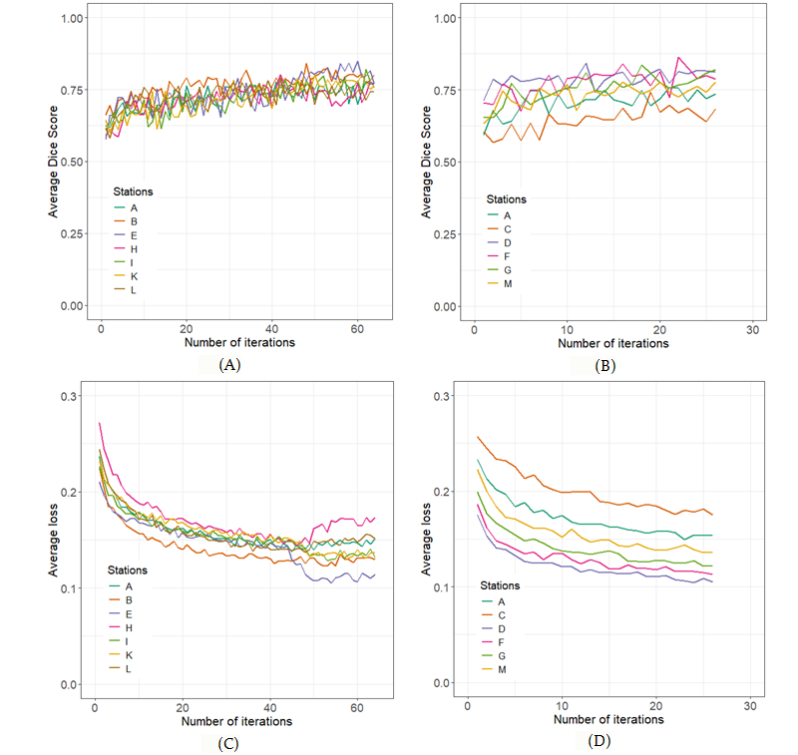
Plots showing the results from training the convolutional neural network on two groups as follows: group 1 (A, B, E, H, I, K, L) and group 2 (A, C, D, F, G, M). (A) Average Dice score per iteration of the model trained on group 1. (B) Average Dice score per iteration of the model trained on group 2. (C) Average training loss per iteration of the model trained on group 1. (D) Average training loss per iteration of the model trained on group 2.

The architectural strategy described above was implemented among ARGOS consortium partners on real-world lung cancer CT scans. For an initial “run-up” of the system, we deployed the abovementioned PHT system across 12 institutions, located in 8 countries and 4 continents. A list of members participating in the ARGOS consortium can be found on the study protocol [26]. In total, 2078 patients’ data were accessible via the infrastructure for training (n=1606) and holdout validation (n=472). For this initial training experiment, the 12 centers were divided into 2 groups. The first, referred to as group A, comprised 7 collaborators, and we were able to reach a total of 64 iterations of model training each with 10,000 steps per iteration. Likewise, group B comprising 6 hospitals was able to train the deep learning model for 26 iterations. It was observed that no significant improvement of the model was observed for both groups after 26th iteration. The results from the proof-of-concept study are shown in Figure 6.

While the training time for the models was similar at each center, how quickly they could be uploaded and downloaded depended heavily on the quality of the internet connection. This meant the entire process was significantly slowed down by the center with the slowest internet.

## Discussion

This study demonstrated the feasibility of a privacy-preserving federated deep learning infrastructure and presented a proof-of-concept study for GTV segmentation in patients with lung cancer. Using the PHT framework, the infrastructure linked 12 hospitals across 8 nations, showcasing its scalability and global applicability. Notably, throughout the process, no patient data were shared outside the participating institutions, addressing significant data privacy concerns. The introduction of a SAS further ensured that model averaging occurred in a secure environment, mitigating potential data leakage issues in FL.

One of the most used methodologies in recent years has been the use of FL for promoting research on privacy-sensitive data. To orchestrate FL on nonstructured data in the horizontal partitioning context, it is essential to develop specialized software for edge computation and technical infrastructures for cloud aggregation. These infrastructures enable federated machine learning (FML) responsibilities to be carried out in a secure and regulated manner. However, only a limited number of these studies have documented the background governance strategies and the ethical, legal, and social implications framework for conducting such studies.

The study presented a novel approach for executing large-scale federated deep learning on medical imaging data, integrating geographically dispersed real-world patient data from cross-continental hospital sites. The deep learning algorithm was designed to automatically delineate the GTV from chest CT images of patients with lung cancer who underwent radiotherapy treatment. The underlying FL infrastructure architecture was designed to securely perform deep learning training and was tested for vulnerabilities from known security threats. This paper predominantly discussed the FL infrastructure architecture and presented a firsthand experience of conducting such studies. The preliminary training of the deep learning algorithm serves as the feasibility demonstration of the methodology, and further refinement is required to achieve acceptable clinical-grade accuracy and generalizability.

The study used an open-source and freely accessible technological stack to demonstrate the feasibility and applicability of federated deep learning. Vantage6, a Python-based FL infrastructure, is used to train and coordinate deep learning execution. TensorFlow and Flask, both open-source Python libraries, are used for the development of the algorithm, subsequently encapsulated within Docker services for containerization purposes. The communication channels between the hospital, central server, and the aggregation node have been secured using Hypertext Transfer Protocol Secure and Secure Hash Algorithm encryption. The hospital sites’ computer systems were based on the Ubuntu operating system and equipped with at least 1 GPU to enhance computational capabilities. The participating centers had the flexibility to choose any CUDA-compatible GPU devices and determine the number of GPUs to use, enabling resource-constrained centers to contribute. However, a limitation exists in terms of computational time due to the synchronous training process being dependent on the slowest participant.

The infrastructure has been tested against known security attacks and as defined by the Open Worldwide Application Security Project top-ten categories [55]. It has been found that the Vantage6 app is impeccable against insecure design, software and data integrity failures, security logging and monitoring failures, and server-side request forgery and sufficiently secured against broken access control, cryptographic failures, injection, security misconfigurations, vulnerable and outdated components, and finally identification and authentication failures. Since the infrastructure is dependent on other underlying technologies like Docker and Flask-API, the security measures in these technologies also affect the overall security of the infrastructure. Additionally, the infrastructure is hosted behind proxy firewalls, adding to its overall security against external threats.

In this study, we implemented a SAS positioned between the data nodes (eg, hospitals and clinics) and the central server. The SAS plays a crucial role in strengthening the privacy and confidentiality of the learning process. The SAS acts as an intermediary that temporarily stores the local model updates from the participating data nodes, ensuring complete isolation from the central server, researchers, and any external intruders. The key benefits of using a dedicated SAS over a random aggregation mechanism in FL are as follows:

- Privacy protection of individual user data and model updates:
  The secure aggregation protocol ensures that the central server only learns the aggregated sum of all user updates, without being able to access or infer the individual user’s private data or model updates.
  By isolating the intermediate updates, the secure aggregation process prevents external attackers from performing model inversion attacks.
- Tolerance to user dropouts:
  The SAS is designed to handle situations where some users fail to complete the execution. In the case of synchronous training, the server stores the latest successful model, enabling data nodes to pick up where they left off instead of restarting from scratch.
- Integrity of the aggregation process:
  The secure aggregation protocol provides mechanisms to verify the integrity of the intermediate models by allowing only the known data nodes to send a model. This maintains the reliability and trustworthiness of the FL system.

FL offers 2 main approaches for model aggregation: sending gradients or weights [56,57]. In gradient sharing, data nodes update local models and transmit the gradients of their parameters for aggregation. Conversely, weight sharing involves sending the fully updated model weights directly to the server for aggregation. Sharing gradients have a higher risk of model inversion attacks. In the study presented here, the data nodes sent model weights instead of model gradients, thus preventing the “gradient leakage” problem. However, weight sharing is not failproof either [58], and the SAS plays a crucial role again in preventing users—internal or external—from accessing the weights from the aggregator machine.

The deployment of the FL infrastructure and training of the deep learning algorithm presented unique challenges that needed to be catered to. Some of them are listed below:

- Heterogeneity across hospitals: Initially, it was not possible to confirm the technology environment at each site. This required significant work to overcome the obstacles connected with each center while deploying a functional infrastructure, good communication, and efficient algorithms.
- Inconsistent IT policies: Standardizing the setup across institutions was hindered by varying IT governance and network regulations in different health care systems across different countries.
- Clinical expertise gap: The predominance of medical personnel over IT specialists at participating hospitals necessitated extensive documentation to ensure clinician comprehension of the FL process.
- Network bottlenecks: Network configurations at participating sites significantly impacted training duration, often leading to delays in model convergence.

The study presented in the paper has identified several areas that require further investigation and improvement. While the findings are valuable, the infrastructure, algorithm, and processes still need to be made more secure, private, trustworthy, robust, and seamless [59]. For example, incorporating homomorphic encryption of the learned models will enhance privacy and provide model obfuscation against inversion attacks. Finally, to further enhance confidence and trust in federated artificial intelligence, it is crucial to conduct additional studies involving a larger number of participating centers and a thorough clinical evaluation of the models.

## Abbreviations

API: application programming interface
ARGOS: artificial intelligence for gross tumor volume segmentation
CNN: convolutional neural network
CT: computed tomography
FedAvg: federated averaging
FL: federated learning
FML: federated machine learning
GPU: graphics processing unit
GTV: gross tumor volume
HIPAA: Health Insurance Portability and Accountability Act
JWT: JSON Web Token
PHT: Personal Health Train
REST: Representational State Transfer
SAS: secure aggregation server
SRE: secure research environment
